# Global threats from invasive alien species in the twenty-first century and national response capacities

**DOI:** 10.1038/ncomms12485

**Published:** 2016-08-23

**Authors:** Regan Early, Bethany A. Bradley, Jeffrey S. Dukes, Joshua J. Lawler, Julian D. Olden, Dana M. Blumenthal, Patrick Gonzalez, Edwin D. Grosholz, Ines Ibañez, Luke P. Miller, Cascade J. B. Sorte, Andrew J. Tatem

**Affiliations:** 1 Centre for Ecology and Conservation, University of Exeter, Penryn Campus, Penryn, Cornwall TR10 9FE, UK; 2 Department of Environmental Conservation, University of Massachusetts, Amherst, Massachusetts 01003-9285, USA; 3 Department of Forestry and Natural Resources, Purdue University, West Lafayette, Indiana 47907, USA; 4 Department of Biological Sciences, Purdue University, West Lafayette, Indiana 47907, USA; 5 School of Environmental and Forest Sciences, University of Washington, Seattle, Washington 98195-2100, USA; 6 School of Aquatic and Fishery Sciences, University of Washington, Seattle, Washington 98195-5020, USA; 7 USDA-ARS Rangeland Resources Research Unit, Fort Collins, Colorado 82001, USA; 8 Natural Resource Stewardship and Science, U.S. National Park Service, Berkeley, California 94720-3114, USA; 9 Department of Environmental Science, Policy, and Management, University of California, Berkeley, California, 94720-3114, USA; 10 Department of Environmental Science and Policy, University of California, Davis, California 95616, USA; 11 School of Natural Resources and Environment, University of Michigan, Ann Arbor, Michigan 48109-1041, USA; 12 Hopkins Marine Station, Stanford University, Pacific Grove, California 93950, USA; 13 Department of Ecology and Evolutionary Biology, University of California, Irvine, California 92697-2525, USA; 14 Department of Geography and Environment, University of Southampton, Southampton SO17 1BJ, UK; 15 Fogarty International Center, National Institutes of Health, Bethesda, Maryland 20892-2220, USA; 16 Flowminder Foundation, SE-11355 Stockholm, Sweden

## Abstract

Invasive alien species (IAS) threaten human livelihoods and biodiversity globally. Increasing globalization facilitates IAS arrival, and environmental changes, including climate change, facilitate IAS establishment. Here we provide the first global, spatial analysis of the terrestrial threat from IAS in light of twenty-first century globalization and environmental change, and evaluate national capacities to prevent and manage species invasions. We find that one-sixth of the global land surface is highly vulnerable to invasion, including substantial areas in developing economies and biodiversity hotspots. The dominant invasion vectors differ between high-income countries (imports, particularly of plants and pets) and low-income countries (air travel). Uniting data on the causes of introduction and establishment can improve early-warning and eradication schemes. Most countries have limited capacity to act against invasions. In particular, we reveal a clear need for proactive invasion strategies in areas with high poverty levels, high biodiversity and low historical levels of invasion.

## Abstract (web summary)

Globalization facilitates the spread of invasive alien species, while environmental change can ease invasion. Here, Early *et al*. identify vulnerable regions globally and evaluate capacity in vulnerable countries to prevent invasions arising from sources such as air travel, horticulture, and pet trade.

## Graphical abstract

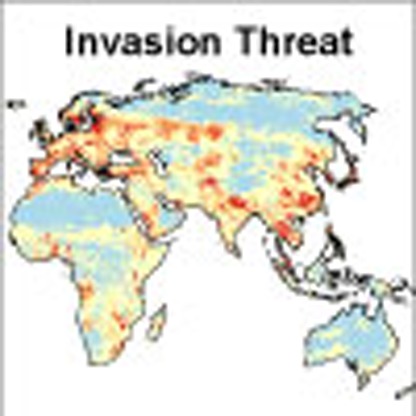

Invasive alien species (IAS) are a primary threat to global biodiversity, economies and human health^1^. The threat of invasion at any given location has been shown to increase with the rate at which IAS propagules are introduced^2^ and the degree of disturbances that promote IAS establishment^3^. Currently, the highest numbers of IAS in the world, the strongest IAS management efforts and the greatest knowledge about the extent of invasions are found in economically developed countries, that is, those with a high Human Development Index (HDI)^4^,^5^. However, the geographical patterns of future invasions is likely to be substantially different from that of today^6^. The intensities and global patterns of introduction and disturbance are changing more rapidly today than at any time during human history^1^,^6^,^7^. Despite these changing threats, national-level legislation to prevent or control IAS has not increased^8^ and, as of 2010, only half of the parties to the Convention on Biological Diversity (CBD) had enacted national legislation relevant to IAS^4^. The regions that will be most exposed to emerging invasions throughout the twenty-first century, and the disparity between IAS threats and capacities to respond to those threats, are therefore poorly quantified.

We provide the first global, spatial forecast of emerging invasions throughout the twenty-first century by analysing spatial data for the above IAS introduction and establishment factors. We also comprehensively assess national IAS response capacities based on reports to the CBD (https://www.cbd.int/reports).

International trade is a primary source of introduction of IAS as stowaways or contaminants in goods and packing materials^2^,^9^,^10^. The pet and plant trades are major sources of animal and plant introductions, due to the frequent escape or release of imported species into the wild^11^, and a primary mechanism for the introduction of insect pests and pathogen contaminants^12^. Transportation as stowaways in passenger planes is a major and expanding source of IAS introductions^9^,^13^,^14^ and marine shipping ports serve as epicentres of invasion^13^,^15^.

Disturbance promotes the establishment of IAS^3^. On a global scale, the most relevant disturbance factors are expansion of agriculture^16^, changes in the composition of native communities as a result of climate change (biome shifts)^17^ and increasing wildfire^18^,^19^.

To demonstrate a strong reactive capacity to control the spread of already-introduced IAS, countries must recognize that IAS threaten that country's environment and economy^20^, have identified IAS already present^21^ and show evidence that IAS policy can be turned into management actions^4^. To have a strong proactive capacity, countries must attempt to prevent the introduction of IAS that are new to that country and control species that are already established and are beginning to emerge as problematic IAS. Thus, demonstrating proactive capacity requires comprehensive border-control policies^22^ and programmes for research, monitoring and public engagement^11^,^23^,^24^ (we classified national response capacities according to the criteria in Supplementary Table 1).

Our analyses of IAS threats suggest that in coming decades, biological invasions will remain high in wealthy and already highly invaded countries. These countries must prepare for a new suite of IAS as climate change disturbs resident ecosystems and introductions of species continue via the pet and plant trade. Less precedented is that IAS will increasingly threaten human livelihoods in low-HDI countries and in the last remaining biodiversity strongholds, where invasions are least well recognized and studied^4^,^5^. Our analysis suggests that current policies in most countries are under-equipped to address emerging threats from IAS, particularly throughout Africa and the eastern hemisphere. Moreover, many of the global biodiversity hotspots that are highly vulnerable to invasion are found in countries that our results suggest have little capacity to respond to IAS (in particular central America, Africa, central Asia and Indochina). Low-HDI countries will particularly require species prioritization and response schemes for IAS introduced via passenger air travel, whose establishment is aided by, and may threaten, agricultural expansion. Early-warning and eradication schemes should be tailored to the factors locally most responsible for introduction and establishment. International sharing of information on IAS and management expertize could greatly help set management priorities in regions that have little capacity to tackle IAS.

## Results

### Global distribution of terrestrial IAS threat

**Figure 1:**
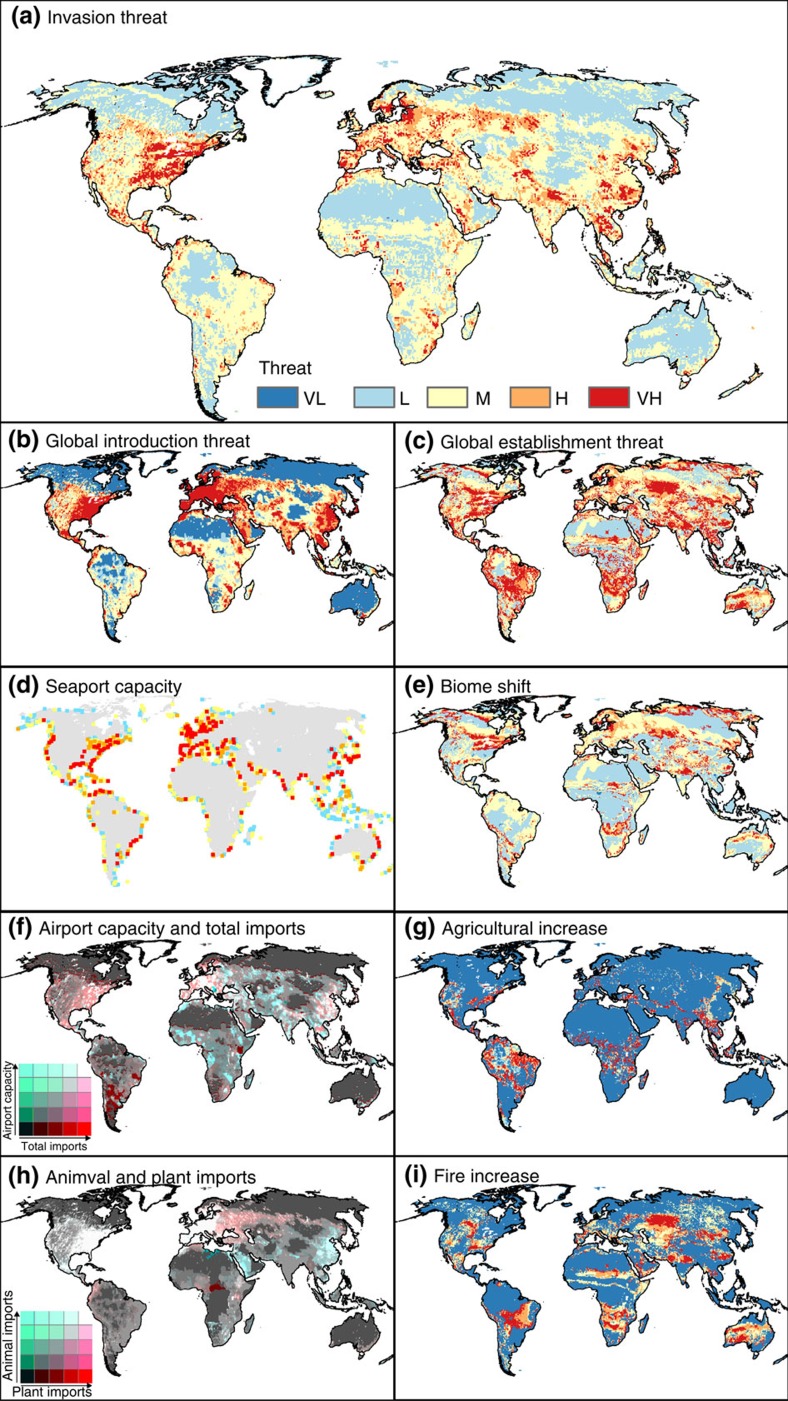
Global invasion threat for the twenty-first century. Airport and seaport capacity, as well as animal, plant and total imports between 2000 and 2009, is combined into global introduction risk. Projected biome shifts and increase in agricultural intensity and fire frequency between 2000 and 2100 (emissions scenario A2) are combined into global establishment threat. Introduction and establishment axes are combined into overall invasion threat (Supplementary Fig. 1). (**a**) invasion threat, (**b**) introduction threat, (**c**) establishment threat, (**d**) seaport capacity, (**e**) climate change-driven biome shift, (**f**) airport capacity and total imports, (**g**) agricultural increase, (**h**) animal and plant imports, and (**i**) fire increase. All maps except (**f**) and (**h**) are displayed using the colour scheme from **a**, which runs from very high (VH; red) to very low (VL; blue). The scale was determined by ranking the threat value in each map grid cell, and binning cells into the following percentiles: 100–90%=very high; 90–80%=high; 80–50%=medium; 50–20%=low; and 20–0%=very low. Maps **b** and **c**, composite introduction and establishment threats, were calculated using the highest value of the constituent factors within each grid cell. Maps **f** and **h** combine the two named threat variables using the colour scheme defined in each panel. In **d**, grid cells containing ports are enlarged for visibility.

The twenty-first century threat of emerging IAS is extensive and distributed globally (Fig. 1). We found that 17% of the global land area (excluding Antarctica and glaciated Greenland) are highly vulnerable to invasion (high and very high categories, Fig. 1a). Although we uncovered threatened areas in some of the most economically developed and currently most invaded regions (for example, western Europe and North America^4^), the threat is also high in parts of low-HDI countries in Africa, South America and Asia (15% of low-HDI countries face globally high or very high threat levels, Fig. 1). Furthermore, 16% of global biodiversity hotspots (Endemic Plant and Bird Areas and WWF's Global 200 Ecosystems) are highly vulnerable to invasion (high and very high categories, Fig. 1).

The distribution and level of threat did not change substantially when alternative predictions of environmental change or establishment factors were applied, but using all passenger air travel rather than only inter-continental journeys changed the spatial extent of some high-threat areas (Supplementary Fig. 1 and Supplementary Discussion).

### Drivers of IAS threat

In high-HDI regions, several introduction vectors for IAS coincide. High levels of general trade and pet and plant imports coincide in Europe, China and the eastern United States (Fig. 1 and Supplementary Fig. 2). Plant and pet imports are particularly common in North America and western Europe, whereas plant imports are the dominant vector in eastern Europe and central Asia, and animal imports are the dominant vector in the Middle East and east Asia (Fig. 1). In high-HDI regions, high IAS threat occurs primarily where introduction vectors coincide with projected climate-driven biome shifts throughout the twenty-first century (Supplementary Fig. 2). Climate change, as expressed through biome shifts (Fig. 1e) and fire frequency changes (Fig. 1i), most directly translates into elevated invasion threat (Fig. 1a) in eastern North America, northern Europe, central and south Asia, polar regions and northern Australia.

In contrast to high-HDI regions, a single introduction vector predominates in low-HDI regions; namely passenger air travel (Fig. 1). Introduction pressure from passenger air travel is now as high in parts of sub-Saharan Africa, the Arabian peninsula, and southeast and south Asia as it is in Europe or North America (light blue or white pockets, Fig. 1f). Seaports make a relatively lower contribution to threats in low-HDI countries than in high-HDI countries (Supplementary Fig. 2). In low-HDI regions, multiple factors that enhance IAS establishment coincide (Fig. 1 and Supplementary Fig. 2). The highest IAS threats in low-HDI regions, and in biodiversity hotspots, occur in regions where globally high levels of passenger air travel overlap with agricultural conversion (Fig. 1 and Supplementary Fig. 2, though notably in the Indo-Gangetic plain IAS introductions are principally driven by general trade).

### National capacities to respond to the IAS threat

**Figure 2:**
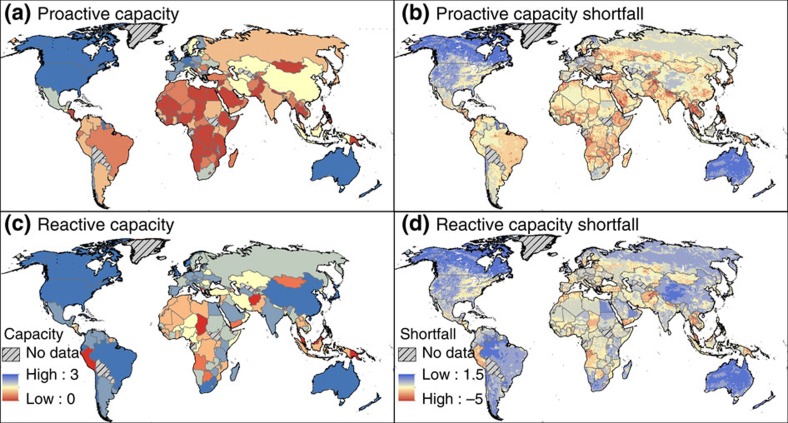
National capacities to respond to the threat of emerging species invasions. (**a**) Proactive capacity: comprehensiveness of measures to prevent the introduction of IAS, and the existence of programmes for research, monitoring, and public engagement to tackle IAS threats. (**b**) the shortfall between threat and proactive capacity calculated by subtracting the threat from Fig. 1a (where VH=5, H=4, M=3, L=2, VL=1) from the capacity value in **a**. Thus negative values (red) indicate the greatest shortfall. (**c**) Reactive capacity: extent of knowledge regarding the current national IAS problem and the degree to which a national action plan exists to prioritise and coordinate IAS management activities. (**d**) the shortfall between threat and reactive capacity calculated by subtracting the threat from Fig. 1a from the capacity value in **c**. Thus negative values (red) indicate the greatest shortfall.

Reactive national policies aimed at managing IAS that are already established and problematic in a given country tend to be more common than proactive policies to detect or counteract the emergence of potential IAS (Fig. 2). Proactive capacities are more advanced in high-HDI countries than low-HDI countries, but surprisingly few high-HDI countries have strong proactive policies, and even fewer countries have both strong reactive and proactive capacities. Areas of Africa, south and central Asia, Indochina, the Balkans, and South and Central America show the greatest shortfall between both types of response capacities and the threat of emerging invasions (Fig. 2). Both knowledge of the current extent of the IAS problem and control efforts for existing IAS (reactive capacities) are relatively poor in much of Africa, and in parts of the Middle East and Central Asia. The proactive capacities of border controls (the most common proactive policy), early-warning systems, research and collaboration are most limited in Africa (Fig. 2 and Supplementary Fig. 3).

## Discussion

The relative threats from IAS that countries around the world will face in the future differ markedly from current threat levels. Although the number of IAS recorded in low-HDI countries currently lags far behind the number in high-HDI countries^4^, the IAS threat to developing economies in the coming decades is set to be far higher than today. IAS are therefore a currently underappreciated and potentially severe element of environmental change in economically developing regions. In such regions, economies and food production systems are often fragile and human populations are particularly vulnerable to food shortages. The major contribution of passenger air travel to introductions into low-HDI regions is particularly concerning because air travel is a major vector of pests and pathogens that can often only survive short journeys^19^ and pose a particular risk to agriculture. Low-HDI regions will thus be faced with mitigating not just the economic, human health and ecological impacts posed directly by environmental changes, but also the effects of increased invasion facilitated by those changes.

It should be noted that twenty-first century threat levels are based on trade and transport levels between 2000 and 2009, to account for time lags between introduction and invasion. As globalization continues apace, introduction pressures on many developing economies may increase even further^6^, worsening the IAS threat. More detailed local analyses would refine understanding of introduction and establishment factors, and improve threat assessments and management tools. However, the global analysis we present here highlights particular areas of vulnerability that should be subsequently addressed by detailed analyses, for example, the prominent role of passenger air travel in low-HDI countries.

Our results should not be taken to suggest that the threats posed by IAS to high-HDI regions will decrease. IAS threats throughout the twenty-first century will be globally high in the world's most developed economies (Fig. 1). High-HDI regions will likely receive proportionally fewer novel introductions than low-HDI regions because many more alien species have already been introduced. Nevertheless, the number of introductions to high-HDI regions has not slowed^9^. Increasing globalization means that new trading relationships are continually forming among countries (adding sources of novel IAS), and environmental change continually increases the ease of establishment^10^.

Aichi Target 9 from the 2011–2020 CBD *Strategic Plan for Biodiversity* states ‘By 2020, invasive alien species and pathways are identified and prioritized, priority species are controlled or eradicated and measures are in place to manage pathways to prevent their introduction and establishment'^25^. We found that most countries have taken steps towards identification and prioritization of some prominent IAS that threaten agriculture, economies or ecosystems, though current management policies only target a handful (≤5) of these IAS for control (reactive capacity, Supplementary Fig. 3). We found that prevention of introduction and establishment (proactive capacity) lags far behind progress towards the reactive CBD goals. The most advanced proactive element is the existence of border controls. The prevalence of border controls is encouraging, as the predominance of passenger air travel as an introduction factor in low-HDI countries suggests that screening these routes is an important component of IAS management. However, in most countries border controls target five species or fewer (Supplementary Fig. 3). Furthermore, although border controls are desirable as preventing introduction is more effective than eradication following introduction^19^, our results are based on trade and transportation values from 2000–2009, which have likely already introduced novel IAS are poised to become problematic. Post-introduction eradication and control of IAS will therefore be important^23^. Eradication therefore requires proactive capacities that support monitoring for early detection of nascent invasions, as well as rapid response to newly discovered populations^23^. Currently, the majority of countries lack the research, management coordination, monitoring of known IAS, early-warning schemes for emerging IAS or public engagement needed in such a campaign (Supplementary Fig. 3), which poses a particularly concerning policy gap.

Strong policies on paper do not necessarily result in effective implementation^22^, though we note that evidence that policies having specific goals, activities and/or outcomes was one of our criteria for reactive capacities (Supplementary Table 1). There is currently no realistic way of evaluating the effectiveness of IAS prevention and management on a global scale, so self-reported capacities remain the most revealing data source. Despite the shortcomings of this approach, CBD reports that contain policies on many elements of IAS response capacities do suggest a higher level of awareness, expertize, legal structure and financial allocation than reports with little such information.

Given the enormous number of known IAS and the unknown number of IAS yet to emerge, rapid evaluation schemes to prioritize responses are crucial^26^. Whilst quantifying introduction pathways and identifying sites that face a high threat of invasion (Fig. 1) are themselves vital to prioritization, knowledge of IAS already present or likely to invade a given country^26^ is equally vital. Lists of harmful alien organisms (LHAOs) aim to prevent the introduction and regulate the management of particularly risky species^21^ (for example, the Australian Weed Risk Assessment scheme^27^), and are one of the most widespread methods for managing IAS threats. To date, LHAOs have largely been used in high-HDI countries and tend to include only a small number of economically damaging pests already present in a country^21^. High-HDI countries contain only a subset of the global range of environmental and socio-economic conditions so LHAOs targeted at these countries are unlikely to capture the IAS that should be prioritized most highly elsewhere. In addition, the current consensus between existing LHAOs is low^21^, with lists likely to underestimate potential IAS due to unknown or underreported species range extents and impacts^28^. These problems highlight the need for improved regional approaches to LHAO development. Neighbouring countries are likely to have similar climates and landscapes, HDI levels, trade and transport connections, agricultural systems and infrastructure. Thus, neighbouring countries are likely to be threatened by the same pool of harmful IAS. Invasiveness elsewhere is one of the most reliable indicators of invasion risk^27^; hence, regional LHAOs can help nations to develop targeted strategies for preventing problematic introductions. A further shortcoming of LHAOs is that the burden of producing and policing their application falls to governments or non-governmental organizations—which could particularly limit the development and use of LHAOs in countries with few economic resources. For species that are deliberately transported (for example, pets and ornamental plants), an alternative could be white listing schemes, which require evidence from those responsible for transportation that a given alien species poses a minimal threat if established^29^. This approach could be particularly useful for countries where ornamental plant and pet trades are major IAS introduction vectors.

Our analysis demonstrates how simultaneously examining introduction pathways and anthropogenic activities promoting invasion can improve LHAOs and risk assessment. This approach can identify IAS, or vectors that favour IAS, with traits that make them more likely to establish and impact native ecosystems^24^. For example, high risk in low-HDI countries could arise from coincidence between intensifying agriculture sectors and high levels of passenger air travel (Fig. 1) that is likely to transport arthropod pests (particularly parts of India, Southeast Asia and southern Africa). Our approach can also be used to improve management: low-HDI countries could prioritize screening of passenger baggage for live plants, fruits or vegetables, which could host crop pests and pathogens. In regions where fire risk is increasing (Fig. 1), agencies could focus on preventing introduction and establishment of disturbance-responsive or fire-adapted plants, which are most likely to arrive via horticulture. Countries with large forestry industries and high levels of cargo imports could prioritize screening of wooden pallets and packaging materials for wood-boring insect stowaways.

In high-HDI countries, newly introduced IAS will add to already high numbers of resident IAS, many of which are increasing the extent of their impacts^30^. Given that climate change-driven biome shifts are the principal establishment factor in high-HDI countries (Supplementary Fig. 2), we suggest that resource management focuses on areas where biome shifts are projected. If an area is transformed into a novel ecosystem^31^, management may need to focus more on the impact of IAS on ecosystem processes than on the presence of IAS themselves.

Combining information on introduction pathways and LHAOs with climate-matching approaches^7^,^32^ would improve priorities for border controls and monitoring for emergence within the threatened country. This information might also prevent the export of IAS to a location where they are anticipated to pose threats, via exit screening of both goods and airline passengers, a policy highly effective in disease management^33^. Progress is being made towards identifying the likely sources of IAS in complicated trade and transportation systems using network theory^34^. These approaches might be particularly useful for low-HDI countries where the main introduction vector, passenger air travel, is already well mapped^7^.

Progress towards prioritization of both species and introduction pathways could be made relatively cheaply through international initiatives for scientific collaboration, data sharing and training. We urge increased exchange of information and skills between regions with a wealth of IAS experts and low-HDI countries that have less expertize. Within-regional collaboration networks are also extremely valuable because of the similar risks faced by neighbouring nations. Regional sharing of data on the status of IAS within a country's borders, outcomes of threat assessments and effective management practices in a timely manner would benefit all nations' IAS control plans. Existing regional efforts such as DAISIE, EASIN, and recent legislation in Europe^35^, the Caribbean Invasive species project, the Australia–Africa Plant Biosecurity Partnership and the Inter-African Phytosanitary Council are good starting points for these efforts.

We were able to compile and analyse the global distribution of invasion threats. Data limitations prevented us from mapping invasion impacts (for example, Australia has relatively low invasion threat (Fig. 1), but IAS in that country have a notably high impact). In low-HDI countries and the biodiversity hotspots often contained within them, even a relatively low global threat may still result in a high impact. Low-HDI countries might be particularly susceptible to invasive impacts because economies and food production systems are fragile and might be easily disrupted by a severe invasion. Also, more of the world's IAS remain to be introduced to low-HDI countries and the biodiversity hotspots contained within them because these countries have experienced relatively little international trade. Current and future increases in trade above historically low trade levels could therefore increase the influx of IAS into low-HDI countries relative to high-HDI countries. Mapping the potential biodiversity, economic and health impacts of current and potential IAS is therefore the next important step in assessing risks, particularly in countries with low HDI and high biodiversity.

## Methods

### A framework for predicting invasions

Our framework evaluates threat, which is defined as exposure to danger, in this case the danger posed by IAS. In this study, threat represents the relative likelihood that IAS are introduced, and become widely established in a given area. The framework is simplified from well-established invasion frameworks^3^,^36^,^37^. We address two stages of invasion. The first is introduction, which includes transport and introduction as defined by Blackburn *et al*.^36^. The second is the naturalization and proliferation of IAS, which broadly corresponds to establishment as defined by Blackburn *et al*.^36^.

### Stage one—introduction

Likelihood of introduction of IAS to a country is driven by the quantity of international trade and the capacity of the transport vectors along which IAS can travel^2^,^10^,^37^. The overall level of international trade in which a country engages corresponds to the quantity of unintentionally introduced IAS^24^,^38^, such as IAS introduced as stowaways, contaminants of crop seed^39^, or as pest and pathogens on food, packaging material or livestock^24^,^40^. The pet and horticultural trades are currently leading vectors of deliberate (as opposed to unintentional) terrestrial IAS introductions. Both trades are increasing globally, are poorly regulated, and can act as vectors for the transported species and their parasites and pathogens^11^,^12^,^39^. In addition to trade and associated air and sea travel, passenger air travel is a major transport vector for IAS (note that introductions via air cargo are accounted for through import value)^15^,^24^. International air travel is a significant factor in the movement of economically damaging pest species: 73% of pest interceptions at the US ports of entry between 1984 and 2000 occurred at international airports, and 62% of intercepted pests at the US ports of entry were associated with baggage^13^, most of which were insects. Substantial illegal imports of fruits, vegetables and animal products via international flights have been recorded^14^. About 52–84% of the genera found in 1 g samples of soil transported in baggage contained species regulated by New Zealand's National Plant Protection Organisation^41^. Transportation of arthropod and microbial pests is particularly likely in baggage due to the degree of protection afforded relative to cargo transportation^41^. Indeed, a substantial number of observed and predicted IAS in Europe have or may travel as stowaways on planes^14^,^15^,^20^.

### Introduction epicentres

Data on trade quantities are typically available at a country level, and passenger numbers are available at an airport level. The final destinations of traded goods, however, are populated areas, in which a high incidence of pet ownership and gardening promotes escape from captivity or cultivation^9^,^11^. Although pests may stowaway in plane cabins and escape once the doors are opened, the bulk of species introduced via passenger air travel are transported onwards in baggage^13^,^41^,^42^. The final destinations of baggage, and IAS introduction locations, therefore correspond to human population density and airport accessibility. Thus, the spatial distribution of introductions correspond to human population density^38^,^43^. A second major set of introduction epicentres are cargo seaports on or directly accessed from the coast, where cargo is unloaded and awaits onward transportation^13^,^44^,^45^.

### Stage two—establishment

One factor promoting establishment is propagule pressure, that is, the number of introduction events and number of propagules introduced^37^. These factors are included in the introduction stage above, and thus we do not repeat them in stage two. Establishment is also promoted by environmental disturbance experienced by the recipient landscape^17^,^31^,^46^,^47^. Disturbance promotes establishment either because native species are poorly adapted to the frequency, intensity or timing of human-mediated disturbances, or because IAS are often adapted to disturbance and thus predisposed for colonization in such environments^17^,^31^.

At a global scale, the most severe disturbances that could promote invasions arise from changing agricultural activities^48^ and fire regimes^18^,^47^, and ecosystem-level impacts of climate change^17^. While many other factors affect the establishment of IAS, their effects are local, idiosyncratic and cannot be quantified on a global scale. Agricultural landscapes are susceptible to invasion due to increased resource availability^31^,^47^ (for example, fertilization or watering), habitat fragmentation^49^, decreased biotic resistance (reduced numbers of competitors, predators and parasites)^49^,^50^ and the predisposition of many of the most damaging IAS to establish in agricultural areas^16^,^39^. Changing fire regimes, including increasing wildfire frequency in many regions^51^, are a major factor changing species compositions and promoting the establishment of IAS. An increase in wildfires can promote invasion by creating germination opportunities and increasing resource availability, but fire suppression can also promote invasion by altering native community structure^18^,^47^,^52^. Warming climates can facilitate invasions by increasing resource availability and invasive habitat suitability (that is, thermal environments that are more suited to invasive than native species, putting native species at a competitive disadvantage)^17^,^53^. Climate change alters the structure of biological communities, that is, the occurrence, abundance and life histories of native species^54^,^55^, which can in turn decrease biotic resistance and facilitate the establishment of alien species^56^,^57^,^58^. These effects will be most severe when climate changes beyond the climatic limits that determine the species assemblage in a given region^59^. Given the coarse spatial resolution of global climate projections, the species assemblages most appropriate for study are biomes, that is, major vegetation types characterized by the same life form. We thus consider climate-driven changes in biome type (biome shifts) to facilitate invasions. Some regions may become less prone to invasion under climate change, however native communities are most often expected to be negatively affected^60^.

Although habitat suitability is important for the establishment of IAS^61^, the effects of habitat suitability could only be assessed by species-specific analyses. Such an analysis is impossible given the paucity of data on the ecological requirements of many known IAS, and since lag phases (below) mean we do not yet know which IAS will have been introduced in the 2000–2010 period for which we quantified introductions.

### Lag phase

The effects of disturbance should be considered throughout the time lag between introduction and emergence of IAS. Choosing an appropriate length of time to study is complicated by high variability in lag phase duration, which can be from tens to hundreds of years^62^,^63^. Consequently, we examine the effects of disturbance throughout the twenty-first century on the establishment of IAS introduced at the beginning of the century.

### Data collation and analysis

All introduction and establishment variables were collated at 0.5° resolution. The area of analysis was limited to the 0.5° terrestrial grid cells for which data for all variables were available (analysis grid), which excluded most oceanic islands and archipelagos. We calculated the area of each grid cell by projecting the analysis grid to a Mollweide Equal Area projection.

We treated the relationships between introduction and establishment factors and invasion as linear, which may not be the case. However, it is not possible to calculate an accurate quantitative relationship between each factor and introduction or establishment of IAS. Given lag phases between introduction and the emergence of invasions, this calculation would require comparison of today's invasions against historical data on introduction and establishment factors. There is no historical period of environmental change that is sufficiently similar to environmental change in the twenty-first century that could be used to do so. Similarly, the geographical patterns, volumes and commodities of today's trade and transportation networks have no historical precedent that could be used to calculate the relationship between introduction factors and invasions. It is also unlikely that each introduction and establishment factor will contribute equally to invasion over the coming decades. For example, stowaways in planes and boats accounted for ∼5–10% of the introductions of terrestrial arthropod species to Europe between 1950 and 2010 (ref. 14). While substantial, stowaway introductions are fewer than the number of alien species introduced as contaminants of commodities^14^. However, the importance of stowaways is likely to be higher in developing economies, as these regions have a higher air traffic:commodity trade ratio than Europe. Given the lack of data for which to calculate numerical relationships between introduction and establishment factors and invasions, the only approach that is currently feasible is to categorize the threat from introduction factors based on relative values among all locations. We measured the threat from establishment factors based on the quantity or likelihood of change in a location over time, and categorized threat levels based on relative values among all locations. This approach allows us to evaluate which of the factors in a given location have particularly high levels relative to other regions and thus are likely to contribute most to the introduction or establishment of IAS (details of threat categorization are below). As more historic data are collated, we hope that improved calibration will permit more precise weighting of introduction pathways (for example, using the approach of Seebens *et al*.^6^).

### Introduction threat factors

Threat of unintentional introduction (for example, via hitchhikers in transported goods) was represented by the mean annual US dollar value of all goods imported by each country from 2000 to 2009. Data were extracted from the United Nations Commodity Trade Statistics database (Comtrade; http://comtrade.un.org). Introduction via horticulture and pet trade was evaluated using the US dollar value of live plants, and live animals not intended for food imported by each country (also using Comtrade data from 2000 to 2009). We predicted the spatial distribution of introductions by distributing import value according to human population density (‘Introduction epicentres', above). We first divided a country's import value by its human population size to calculate a per capita import value. We then multiplied per capita import values by population density in each analysis grid cell to yield the mean import dollar value per square metre. Population data were obtained from the Global Rural–Urban Mapping Project^64^ at 30 arcsec resolution, and values summed within each 0.5° grid cell.

Threat of introduction via passenger air travel was calculated using the estimated number of passengers arriving at all airports located in cities with populations >100,000 in 2010 (ref. 65). The data set comprises the number of passengers for which each airport is their final destination, and the number of these passengers that began their journey at each alternative airport. Travel between airports was assessed using both direct flights and journeys that required one or two stopovers. Owing to the ‘hub-and-spoke' format of the global air travel network, this form of ‘origin–final-destination' travel has been identified by epidemiological studies to be the most important in the spread of vector-borne diseases^7^. These airports act as hubs for inter-regional travel demands, and travel between them represents the most important air-based introduction routes for vector-borne diseases, and likely IAS^7^. Our main analysis used the number of arriving passengers that had originated from an airport on another continent, as inter-continental IAS introductions are result in a greater number of invasions and problematic invasions in terrestrial systems^66^,^67^. However, we also performed a supplementary analysis using passenger arrivals from all origins (Supplementary Fig. 1). Each grid cell was considered to be in the area of influence (that is, receive passengers from) of the nearest airport, on the basis that travellers would choose the nearest airport to their destination. However, we assumed that travellers would not cross international borders to reach an airport, with the exception of the European Schengen zone, in which international travel is unrestricted. Thus, outside the Schengen zone, areas of influence were limited to the country in which the airport was situated. In addition, when several airports were separated by distances <50 km and not separated by an international border or sea, airports were grouped, areas of influence combined and their passenger numbers summed. We used human population density within the area of influence of each airport or group of airports to calculate the relative introduction per threat person per square metre, in each analysis grid cell globally.

Likelihood of introduction at port locations was calculated from the cargo traffic (port volume data, in metric tons) of each port listed in the World Port Index compiled in 2003 (ref. 68). We assigned the introduction potential from each port to the coastal grid cell it intersected or to which it was closest.

For each introduction factor, we ranked grid cells globally and binned them into relative threat categories so that the geographic area of the analysis grid included in each category fell into the following percentiles: 100–90%=very high; 90–80%=high; 80–50%=medium; 50–20%=low; and 20–0%=very low. Note that for some introduction threat factors, more than 20% of grid cells had a threat of 0 and were included in the very-low-threat category. Binned threat categories at the low end of the risk spectrum might not exactly reflect the above percentiles.

### Establishment threat factors

Biome shifts due to climate change were analysed using the MC1 dynamic global vegetation model, comparing climate between 1961–1990 (observed) and 2071–2100 (projected)^69^. Climate conditions were projected using three GCMs (CSIRO Mk3, HadCM3 and MIROC 3.2) and the B1, A1B and A2 emissions scenarios. Grid cells were assigned a biome shift value between 0 and 1 based on the level of confidence that their biome will change, that is, the proportion of combinations of GCMs and emissions scenarios in which the biome is projected to change^69^. Threat was classified using the following confidence values: >0.9=very high; 0.8–0.9=high; 0.5–0.8=medium, 0.2–0.5=low, and <0.2=very low.

Change in wildfire frequency due to climate change was calculated by comparing fire observations from 1951 to 2000 (ref. 70) against 2051–2100 fire projections from the MC1 dynamic global vegetation model^69^,^71^. The MC1 model was run using three GCMs (CSIRO Mk3, HadCM3 and MIROC 3.2) projected separately for A2 and B1 emissions scenarios. We categorized the threat of fire increase by ranking grid cells in which fire frequency change was positive and binning into the same area percentiles as were used for introduction threat factors. All zero or negative values were assigned ‘very low' threat. Note that change in wildfire frequency was also one of the driving factors in the biome shift analysis^69^. We include fire as well as biome shifts as the important direct effects of fire on IAS would otherwise be excluded. Moreover, by classifying invasion threat based on the threat factor with the highest value for each cell (see below) we avoid the effects of replicating threat factors.

As a supplementary analysis, we also evaluated the impact of fire decrease. We categorized the threat of fire decrease by ranking grid cells in which fire frequency change was negative and binning into the same area percentiles as were used for introduction threat factors. All zero or positive values were assigned ‘very low' threat (Supplementary Fig. 1).

Twenty-first century agricultural (crop or grazing) land was predicted using IMAGE 2.2 class one classifications ‘cropland', ‘fallow land' and ‘grassland'^71^,^72^ under greenhouse gas emissions scenarios A2 and B1. Agricultural change was evaluated against a baseline of the number of decades between 1970 and 2010 that each grid cell was classed as agricultural. The baseline was subtracted from the number of decades that the grid cell was agricultural between 2060 and 2100 to calculate an agricultural index between −4 and 4 (ref. 72). This index summarizes both the intensity of agricultural activity and its likelihood, that is, a grid cell predicted to be used for agriculture in a single decade is only marginally or transiently likely to be agricultural. We used index values to classify threat from agricultural conversion as follows: 4=very high; 3=high; 2=medium; 1=low; and <1=very low.

### Combining factors to calculate threat

We required both introduction and establishment factors to be high in order for a location to be exposed to high threat. We classified each grid cell according to the highest level of threat posed by any introduction and establishment factor, following the scheme in Supplementary Fig. 4.

This approach assumes that threat factors are redundant, rather than additive. For example, if a grid cell has high establishment threat due to agricultural conversion, and medium threat due to fire increase, the cell is assigned ‘high' threat. We also investigated how these results would differ if a consensus approach were taken. That is, we varied the number of introduction and establishment factors in a grid cell that would have to be ‘very high' or ‘high' to classify the grid cell's overall threat level as such. We then examined the spatial patterns of variation in high and very high threat areas that would result from different consensus approaches (Supplementary Fig. 5).

The main threat assessment was performed using fire increase and agricultural increase under the A2 emissions scenario, but we also conducted supplementary analyses using fire decrease under the A2 scenario, and fire and agricultural increase under the B1 scenario. To evaluate how robust our results were to differing methodologies, we evaluated the consistency in our predictions. Consistency was the proportion of the very high or high+very high threat cells in our main threat assessment that would be evaluated similarly if the alternative threat categorization was used (Supplementary Table 2).

We evaluated invasion potential globally, and in regions of particular interest: (1) G200, the terrestrial Global 200 Ecosystems, which represent the most ‘outstanding' examples of each major habitat type; (2) endemic areas, the Endemic Bird Areas and Centres of Plant Diversity^73^,^74^; and (3) highly and poorly economically developed countries according to 2005 values of the HDI^75^ (HDI>0.8 and HDI<0.5, respectively). The G200 and endemic areas are collectively termed ‘biodiversity hotspots'. See Supplementary Fig. 6 for maps of analysis regions.

### Map of capacity for dealing with IAS threats

To evaluate each country's capacity to respond to IAS threats that may emerge during the twenty-first century, we analysed the fourth and fifth national reports on the implementation of the CBD (submitted between 2008 and 2014, 181 reports in total). These reports assess each country's progress towards the 2010 Biodiversity Target (of which Goal 6 is ‘Control threats from invasive alien species'), and/or towards targets in the 2011–2020 CBD *Strategic Plan for Biodiversity*. These reports are the most standardized documents available for comparing results between countries. As the United States is not a party to the CBD, we evaluated its capacity based on the US National Invasive Species Council's *Invasive Species Management Plan*^76^.

We considered a country's capacity for reactive and proactive responses to IAS. Reactive response capacity was demonstrated by the extent of knowledge regarding the current national IAS problem and the degree to which a national action plan existed to prioritize and coordinate IAS management activities. A strong reactive response capacity demonstrates existing IAS threats are on the national biodiversity or economic agenda and that a country possesses the expertize, resources and willingness to mitigate the damage caused by IAS. Proactive response capacity was demonstrated by the comprehensiveness of measures to control the introduction of IAS, and the existence of programmes for research, monitoring and public engagement to tackle IAS threats. A strong proactive response capacity demonstrates that a country is monitoring for emerging IAS problems and the possibility of prevention or early containment of emerging invasions is relatively high. We included efforts led by governments, research institutions, governmental organizations or private institutions with a duration of more than one year. See Supplementary Table 1 for the evaluation framework.

The track record of 181 countries on combatting IAS—policies, resources and legislative capacities—indicates potential to combat IAS effectively in the future. Our approach is designed to distinguish countries that are ill-prepared and potentially well-prepared, rather than separate out the countries that are extremely well-prepared.

We developed this framework using CBD reports from 15 countries drawn from across the world that have a representative range of economic development levels (which also reflects different levels of IAS policies and awareness^4^,^5^). These countries were Albania, Austria, Bangladesh, Canada, Indonesia, Iran, Jamaica, Jordan, Lesotho, Madagascar, Slovenia, Suriname, Switzerland, Thailand and Zimbabwe. For these countries, the first three authors reached a consensus of the most appropriate interpretation of capacity. Each CBD report was then reviewed by two independent researchers, who underwent training by reviewing the 15 reports already evaluated and comparing their assessments with the consensus assessments. Discrepancies between the two reviewer assessments were identified, discussed between reviewers and the authors, and reconciled. We did not measure uncertainty in our estimates, but instead minimized the possibilities for multiple interpretations of the information in reports. The criteria themselves (Supplementary Table 1) were designed to be as simple as possible, requiring only the amount of information usually available in the CBD reports, so as to minimize the subjectivity of the process. We do not believe attempting to quantify uncertainty in our interpretations is appropriate, because the degree of uncertainty itself would be subjective, and so would not improve understanding of the precision of our results.

### Data availability

The data that support the findings of this study are available from the corresponding author on request, except for data on change in wildfire frequency and biome shifts, which are available from P.G., and on passenger air travel, which are available from A.J.T.

## Additional information

**How to cite this article:** Early, R. *et al*. Global threats from invasive alien species in the twenty-first century and national response capacities. *Nat. Commun.* 7:12485 doi: 10.1038/ncomms12485 (2016).

## Supplementary Material

Supplementary Information Supplementary Figures 1-6, Supplementary Tables 1-2 and Supplementary Discussions and Supplementary References
