# Supplementary material for: Global threats from invasive alien species in the twenty-first century and national response capacities
Source: Nat Commun. 2016 Aug 23;7:12485. doi: 10.1038/ncomms12485 (PMC4996970; doi:10.1038/ncomms12485)
Supplement: Supplementary Information — Supplementary Figures 1-6, Supplementary Tables 1-2 and Supplementary Discussions and Supplementary References [file ncomms12485-s1.pdf]

Supplementary Figures

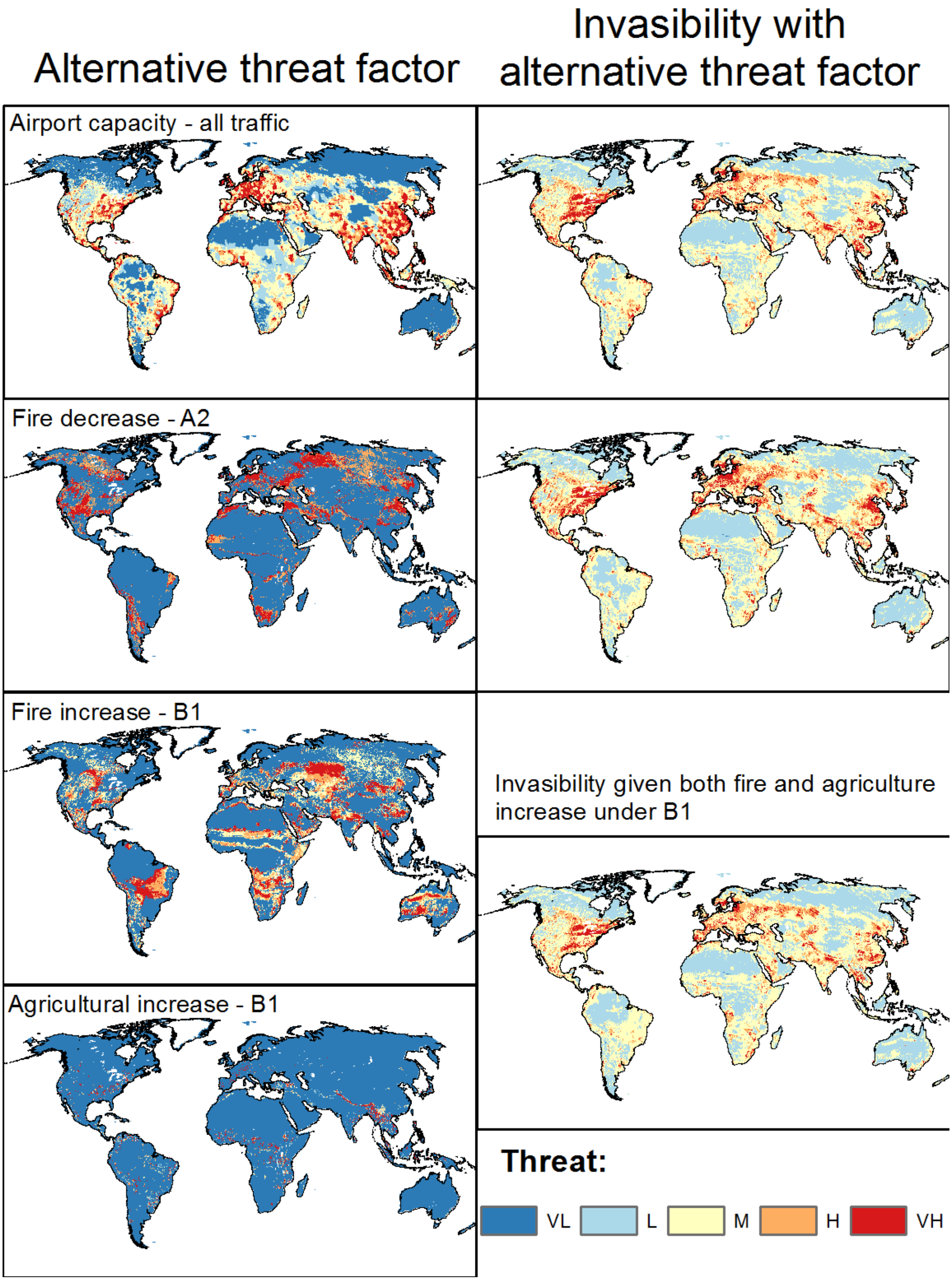

Supplementary Figure 1. The outcomes of alternative methods for calculating threat categories and the resulting invasibility assessments.

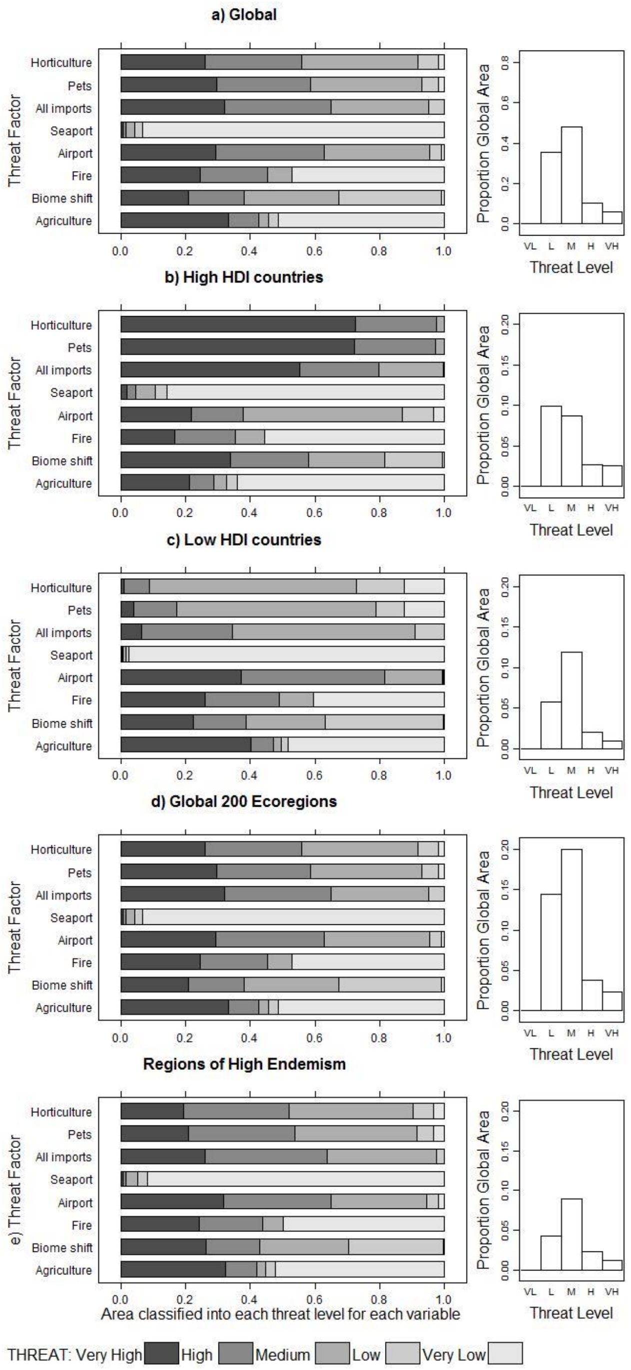

Supplementary Figure 2. Contribution of each introduction and establishment factor to the invasion threat in different regions. Left hand panels: the factors driving the categorization as at High or Very High threat. Within the High and Very High threat areas of the named region, the bars show the proportion of cells within each region that are classed as VH/H/M/L/VL threat according to each factor individually. Right hand panels: histograms of the proportion of the global area that is categorized into each of the threat categories within each of the named regions.

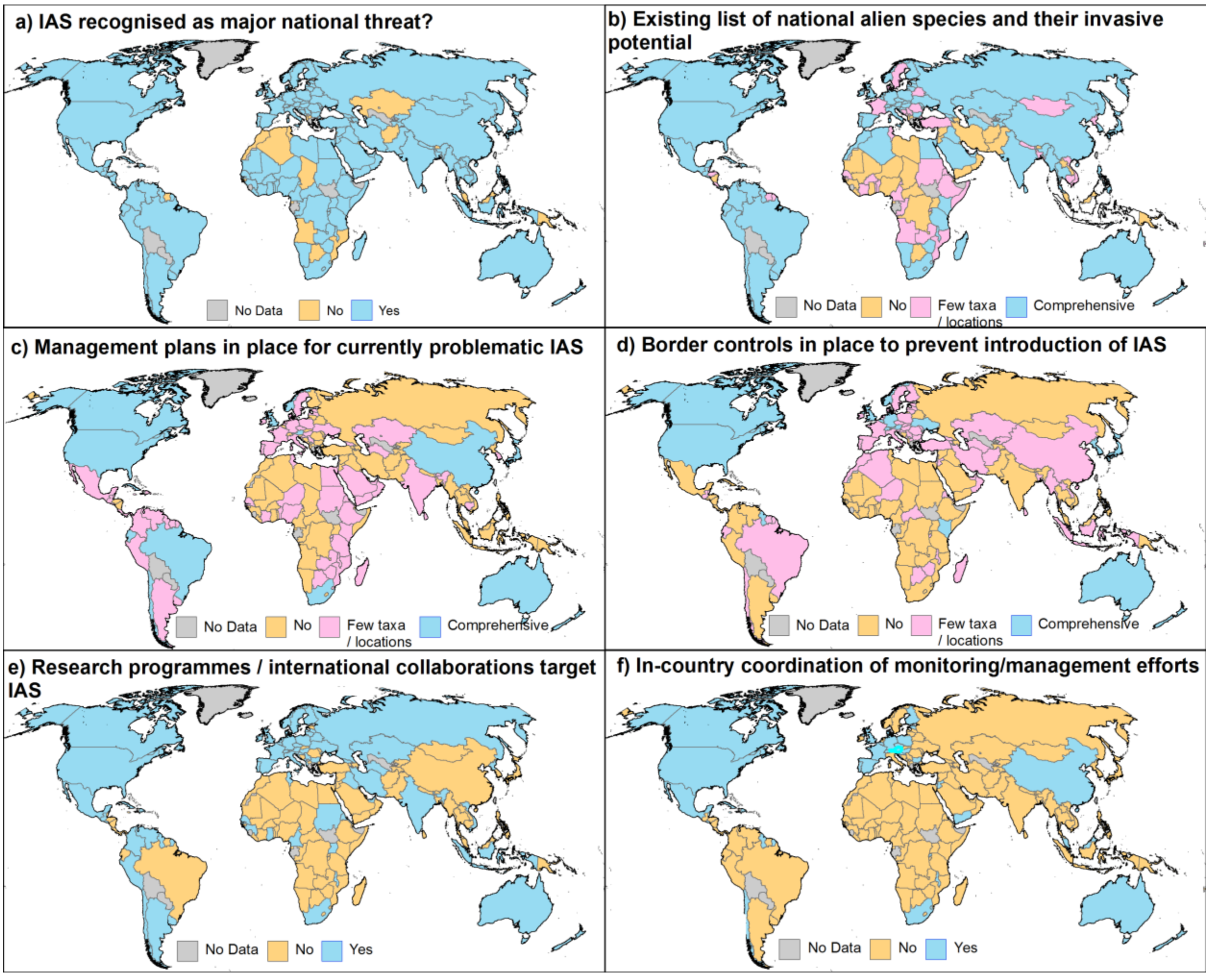

Supplementary Figure 3. The individual elements that contribute to reactive and proactive capacity to respond to IAS threats in Fig. 3. Reactive policies: (a) IAS recognised as a major threat to national biodiversity, (b) a list of national alien species and their invasive potential has been prepared, (c) management plans in place for currently problematic IAS. Proactive policies: (d) measures are in place to prevent introduction of new IAS, (e) active research programmes into IAS and international collaborative efforts to prevent or mitigate IAS impacts are in place, (f) in-country awareness-raising programmes and coordinated efforts to monitor the spread of known IAS and the emergence of new IAS are underway.

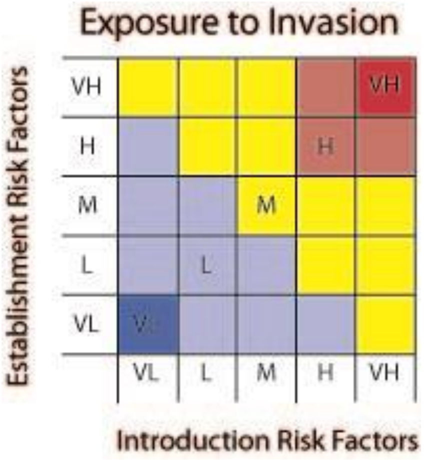

Supplementary Figure 4. Method for combining threat levels from the pooled introduction threat and pooled establishment threat axes to form an overall assessment of vulnerability to invasion for each grid-cell.

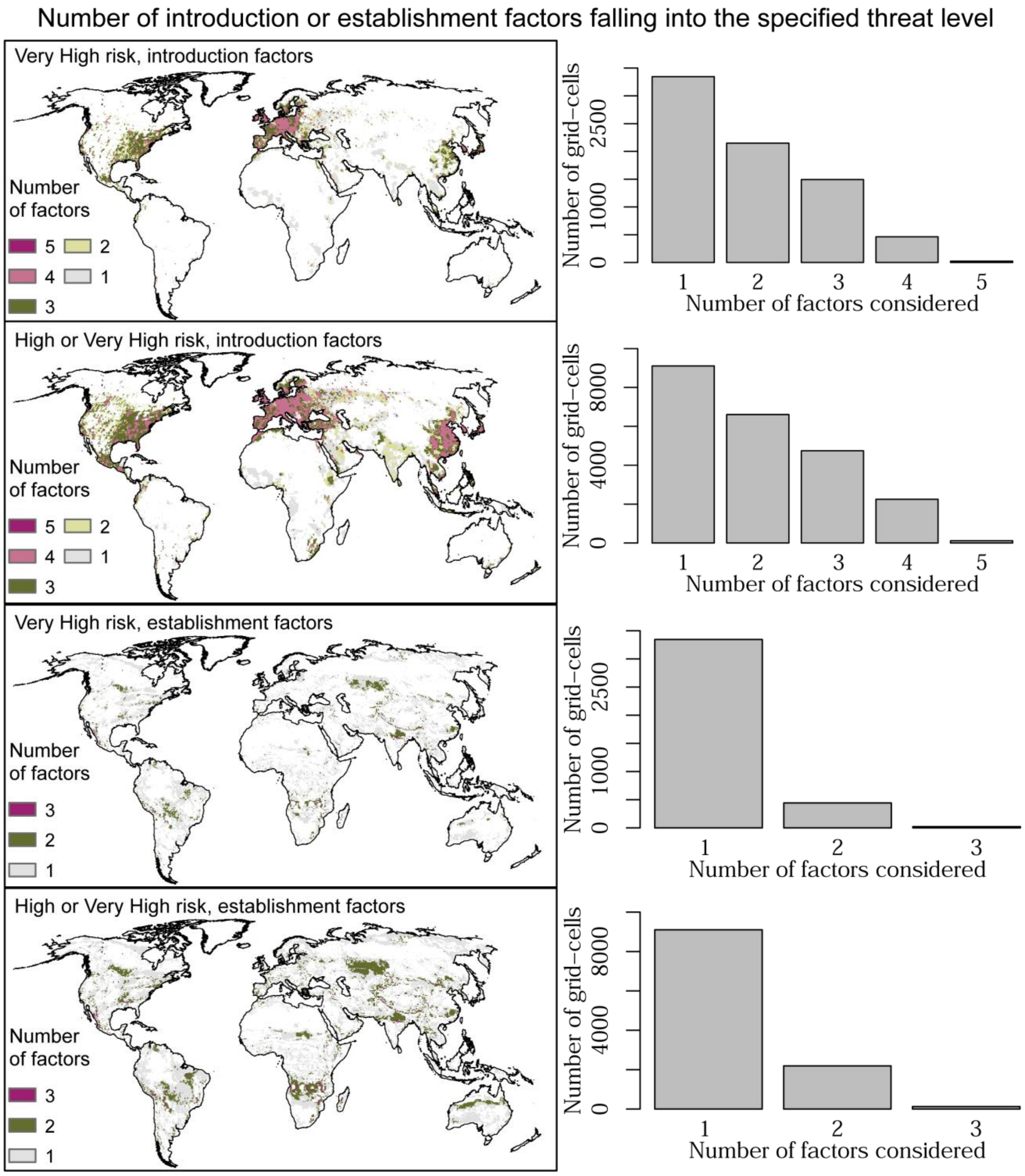

Supplementary Figure 5. The impacts of using a consensus approach to combine introduction and establishment factors to assess invasion threat. The maps show the number of introduction or establishment factors in each grid-cell that are classified as High or Very High threat. The corresponding histograms show the number of grid-cells that would be evaluated to be at High/Very High or Very High threat using the highest-value threat factor in a grid-cell (one factor considered, the approach that was used in the main threat assessment), or on the 2nd or lower highest-value factor (i.e. if two or more factors were required to be at High or Very High threat).

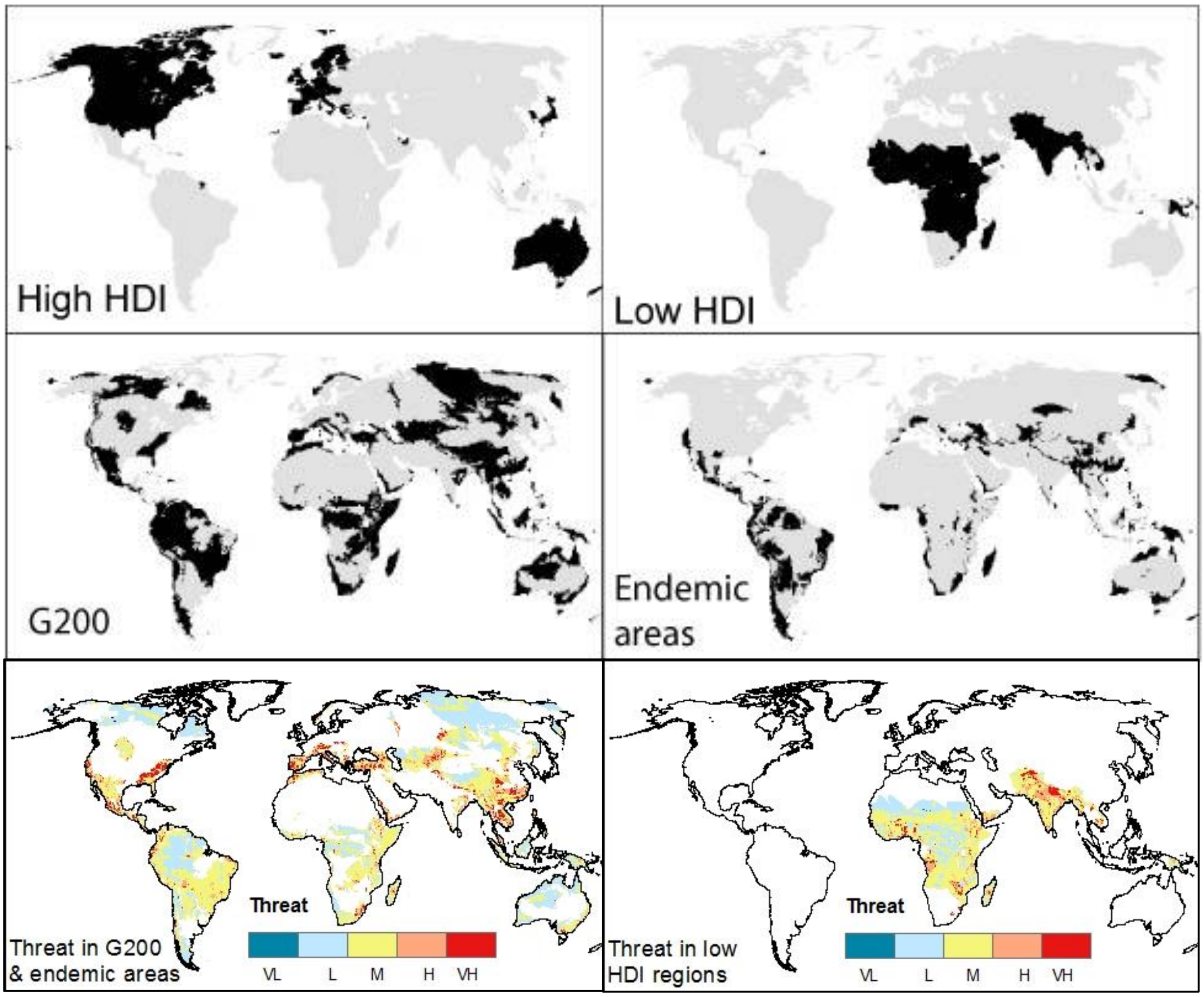

Supplementary Figure 6. Global analysis region (grey) with areas included in each regional analysis (black), and threat levels of future invasion (from fig. 1) in G200 and endemic areas (biodiversity hotspots), and in low-HDI regions.

Supplementary Tables

Supplementary Table 1. Criteria for categorising each country's capacity to deal with emerging IAS threats

Action	Type of capacity	Categories (numerical value summed to evaluate reactive or proactive capacity)
(a) IAS considered a major threat to national biodiversity or economy	Reactive	1. Yes: The text must specifically mention a perspective on IAS threats within a given country, not use generic language or refer to IAS in general.<br>0. No: does not meet the criteria for 'yes'.
(b) A list of current and/or potentially problematic IAS has been developed	Reactive	1. Comprehensive: systematic listing procedure covering several taxa and the whole country. This is evidenced by the country having compiled a list of current problematic IAS that contains >5 species and in a table or grouped into a list specifically identified as problematic IAS (i.e. some IAS are not simply mentioned in the text in an *ad hoc* way).<br>0.5. Limited: some high profile species mentioned in the text, but no evidence of data having been gathered widely.<br>0. No: none of the above.
(c) Engages in management of existing IAS problems	Reactive	1. Comprehensive: management strategies covering several taxa throughout country. This is evidenced by the existence of policy, legislation, resources, or informal (but well-specified) measures to manage existing IAS. Management must be in place or have been enacted; management that is part of a strategy not yet in place would not be included. Specific goals, projects, and/or outcomes listed. Management must be undertaken nationally, or has a geographical scope that addresses the points of introduction and establishment of targeted species. Management could include general measures that are prepared to target any problematic species, species that are part of an international LHAO (e.g. the IPPC pest list), or >5 IAS identified as problematic in the country.<br>0.5. Limited: few species targeted, efforts restricted to a few regions. This is evidenced by the existence of policy, legislation, resources, or informal (but well-specified) measures to manage existing IAS. Management must be in place or have been enacted; management that is part of a strategy not yet in place would not be included. Specific goals, projects, and/or outcomes listed. Management has been attempted for ≤5 species.<br>0. No (includes management plans in development but not yet deployed).
(d) Measures are in place to control introduction of potential IAS	Proactive	1. Comprehensive: inspection procedures in place to identify and quarantine or prohibit entry of a comprehensive list of known/potential IAS. This is evidenced by the existence of policy, legislation, resources, or informal (but well-specified) measures to control the introduction of species (e.g. quarantine, prohibition, inspection, black lists). Management must be in place or have been enacted; management that is part of a strategy not yet in place would not be included. Management could include general measures that are prepared to target any potential IAS, IAS that are part of an international LHAO (e.g. the IPPC pest list), or >5 IAS identified as potentially problematic in the country.<br>0.5. Limited: to a few taxa and border locations, or within country only. This is evidenced by the existence of policy, legislation, resources, or informal (but well-specified) measures to control the introduction of species, but these measures have one or both of the following limitations: (i) restrict introduction between parts of the same country rather than introductions from outside the country; (ii) they target ≤5 species. Management must be in place or have been enacted; management that is part of a strategy not yet in place would not be included.<br>0. No (includes introduction measures in development but not yet deployed).
(e) Active research into IAS or international coordination of control efforts	Proactive	1. Yes: outreach, education or research activities related to IAS exist. These projects are long-term (>1 year) with specific goals, projects, and/or outcomes listed, and the responsible parties are listed in the CBD document. Efforts must be in place or have been enacted; efforts that are part of a strategy not yet in place would not be included. In Europe, all contributors or partners of DAISIE are considered to be classed as 'yes'.<br>0. No: includes situations where one-off and short-term events (≤1 year) are mentioned, or activities are described very vaguely (e.g. 'engaging in regional dialogue').
(f) Efforts to monitor IAS emergence or expansion within country	Proactive	1. Yes, existence of the following: long-term programs to raise public awareness, dissemination of resources for species identification and management, facilities for reporting emerging or spreading invasions. These projects are long-term (>1 year) with specific goals, projects, and/or outcomes listed, and the responsible parties are listed in the CBD document.<br>0. No: includes situations where one-off and short-term events (≤1 year) are mentioned.

Supplementary Table 2. Consistency between the threat assessment presented in the main text and the results obtained using alternative methods for categorizing individual threat factors.

Alternative threat classification	Consistency with Very High areas	Consistency with High or Very High areas
Airport capacity – all traffic	0.40	0.51
Fire decrease – A2	0.73	0.75
Fire and agricultural increase – B1	0.70	0.80

Consistency is the fraction of the areas evaluated in the main threat assessment as Very High or High + Very High, that would be evaluated similarly if the alternative threat classification was used.

Supplementary Discussion

Alternative threat classifications

When an alternative greenhouse gas emissions scenario was used (B1, in which warming is less than under the A2 scenario) and fire decrease was considered as an establishment factor rather than fire increase, consistency with the original threat classification was high. 70% or more of the original High and Very High threat areas remained classified as such (Supplementary Table 1). Under the B1 emissions scenario, fire was the greatest relative establishment factor globally, due to the changed spatial pattern of fire outbreak (Supplementary Fig. 5).

However, when passenger travel between any parts of the world, rather than solely between continents, was considered, consistency was 40-51%. Major discrepancies occurred in Africa, where the locations of High and Very High threat hotspots were similar, but the spatial extent of each hotspot was reduced. A similar, but less pronounced, pattern occurred in South America. Elsewhere, the threat levels did not decrease (Supplementary Fig. 5). Given the propensity of inter-continental transportations to result in the emergence of new IAS[1, 2], and that the locations of High and Very High threat hotspots are stable, we consider the threat presented in Figure 1 (main text) to be the most informative map of the threat of emerging invasions. Nonetheless, intra-continental transportation could enhance the spread of IAS already found in a continent, and this threat needs to be tackled most forcefully in the HVH areas highlighted in Supplementary Fig. 5.

Supplementary Fig. 6 shows the differences in threat categorization that would result if we used the highest threat levels of two or more of the introduction or establishment factors in a grid-cell in order to classify overall threat (rather than using the single highest threat level within both the introduction and establishment factors). The levels of multiple introduction factors were high in Western Europe, North America, and China, whereas in much of Africa and South America, threat from a single introduction factor was responsible for the classification of a grid-cell as High or Very High threat. High levels of the establishment factors were more dispersed, meaning that in most locations, a single introduction factor was responsible for the classification of a grid-cell as High or Very High threat. Notable locations in which multiple establishment factors coincided are in the Siberian Plain, and humid subtropical parts of Africa and India.

Supplementary References

1. Ricciardi A, Simberloff D. Assisted colonization is not a viable conservation strategy. *Trends Ecol Evol* **24**, 248-253 (2009).

2. Mueller JM, Hellmann JJ. An Assessment of Invasion Risk from Assisted Migration. *Conserv Biol* **22**, 562-567 (2008).
